# Assessment of Intratumoral Micromorphology for Patients with Clear Cell Renal Cell Carcinoma Using Susceptibility-Weighted Imaging

**DOI:** 10.1371/journal.pone.0065866

**Published:** 2013-06-06

**Authors:** Jie Chen, Jiule Ding, Yongming Dai, Wei Xing, Jun Sun, Zishu Zhang, Yang Xuan, Vasuki Pilli, E. Mark Haacke, Jiani Hu

**Affiliations:** 1 Department of Radiology, Affiliated Third Hospital of Suzhou University, Changzhou, Jiangsu, China; 2 Department of Radiology, Wayne State University, Detroit, Michigan, United States of America; 3 Siemens Healthcare China, MR Collaboration NE Asia, Shanghai, China; 4 Department of Radiology, University of Michigan, Ann Arbor, Michigan, United States of America; Wayne State University, United States of America

## Abstract

**Background:**

Multiple treatment options exist for the management of renal cell carcinomas. Preoperative evaluation of clear cell renal cell carcinoma (CRCC) grades is important for deciding upon the appropriate method of therapy. We hypothesize that susceptibility weighted imaging (SWI) is sensitive enough to detect intratumoral microvessles and microbleeding in renal cell carcinoma, which can be used to grade CRCC.

**Material and Methods:**

Retrospective reviews of 37 patients with pathologically proven CRCCs were evaluated. All patients underwent SWI examinations. The characteristics of intratumoral susceptibility signal intensity (ITSS) includes the likelihood of the presence of ITSS, morphology of ITSS, dominant structure of ITSS and ratio of ITSS area to tumor area, which were all assessed on SWI. The results were compared using the nonparametric Mann-Whitney test.

**Results:**

ITSS was seen in all patients except 4 patients with low-grade CRCCs. There was no significant difference between low and high-grade CRCCs when looking at the likelihood of the presence of ITSS. There was a significant difference in the mean score of dominant structures between low and high-grade CRCCs. Specifically, more dominant vascular structures and less hemorrhage were seen in low-grade tumors (2.15±1.05) compared to high-grade tumors (1.27±0.47) (*P*<0.005). The ratio of ITSS area to tumor area was also significantly higher for the high-grade group (1.55±0.52) than that for the low-grade group (0.88±0.43) on SWI (*P*<0.005).

**Conclusion:**

SWI is useful for grading CRCCs.

## Introduction

Renal cell carcinoma (RCC) is the most common type of primary renal malignancy in adults and is responsible for approximately 85%∼90% of all cases [1]. Clear cell renal cell carcinoma (CRCC) is the most common histological subtype of RCC, accounting for 75–88% of RCCs [2], [3]. The nuclear grade of CRCC is an independent predictor of prognosis and can help assess tumor aggressiveness [4], [5].

Correlations between pathological grades of CRCC and tumor size have been reported in previous studies [6]–[8]. However, the correlation between tumor size and pathological grade is still controversial. Kitagawa et al. [9] reported that no correlations between tumor size and Fuhrman nuclear grade were present.

Susceptibility weighted imaging (SWI) is a relatively new technique that combines magnitude and phase information to improve the sensitivity for visualizing paramagnetic materials such as deoxygenated hemoglobin and hemosiderin. Because of its sensitivity to susceptibility effects, SWI can noninvasively visualize microvenous structures and blood products that are not visible in conventional MR. Many previous studies have reported that microvenous structures showed a fine linear or conglomerated linear hypointensity, while hemorrhage shows a dot-like hypointensity with or without conglomeration [10], [11]. The distribution and count of microvenous structures and hemorrhage have been shown to correlate with tumor grade in the brain [10], [11].

The use of SWI in the abdomen had been limited by technical issues, including breathing and motion artifacts from long acquisition times. Recently, a few studies on abdominal SWI have been conducted with the development of a new multi-breath-hold two dimensional (2D) GRE based SWI (Work in progress sequence, [WIP#608], Siemens Healthcare) [12]–[14]. To our knowledge, the correlation between pathological grades and ITSS on SWI in CRCCs has not been assessed. This study is aimed to evaluate the feasibility of utilizing SWI to characterize intratumoral susceptibility signal intensity (ITSS) and exploring the role of SWI in grading CRCCs.

## Materials and Methods

### Ethics Statement

All research procedures were approved by the Institutional Review Board of Affiliated Third Hospital of Suzhou University and were conducted in accordance with the Declaration of Helsinki. Written informed consent was obtained from all patients.

### Subjects

A total of 38 patients were enrolled in this study. Inclusion criteria included: patients must have underwent MR imaging for evaluation of renal masses, radical nephrectomy and had pathological confirmation of CRCCs. One patient, who’s MRI had exhibited breathing artifacts, was excluded from the study. In the end, 37 patients (23 men and 14 women; ranging 21–77 years; median age of 56 years) were included in this study.

### MRI examination

All examinations were performed on a 3T scanner (MAGNTEOM Verio, Siemens Healthcare, Erlangen, Germany) using a standard 12-channel phase array body-matrix coil.

The protocol for the MRI examination given to all the patients was: (a) coronal breath-hold half acquisition single-shot turbo spin echo (HASTE) T2-weighted imaging (T2WI) (TR/TE, 800/91 ms; field of view, 380 mm×380 mm; matrix size, 117×256; slice thickness, 4 mm; gap 1.95 mm; flip angle, 160°; bandwidth, 781 Hz/pixel); (b) transversal GRE T1-weighted imaging (T1WI) (TR/TE, 161/2.5 ms; field of view, 285 mm×380 mm; matrix size, 180×320; slice thickness, 5 mm; slice gap 1.0 mm; flip angle, 70°; bandwidth, 270 Hz/pixel); (c)transversal HASTE T2WI (TR/TE, 700/96 ms; field of view, 285 mm× 380 mm; matrix size, 168×320; slice thickness, 5 mm; gap 1.0 mm; flip angle, 150°; bandwidth, 488 Hz/pixel); and (d) transversal 2D breath-hold SWI (TR/TE, 162/10.3 ms; field of view, 285 mm×380 mm; matrix size, 187×384; slice thickness, 5 mm; gap 1.0 mm; flip angle, 20°; bandwidth, 620 Hz/pixel).

The order of all transversal sequences performed was T1WI, T2WI and SWI. All slices were obtained in slice-match mode by choosing the option of “copy the reference” on an operation workstation. 2D SWI was performed through 2∼3 breath-holds, each of which lasted 12∼16 seconds including a 5 second break, and the total acquisition time was around 1 minute depending on the respiratory condition of the patient. Suspended respiration was most reproducible at end expiration with a brief coaching session prior to the examination.

2D SWI post-processing was done inline and consisted of the following steps: 1) the k-space complex data of each channel from the 12 channel body-matrix coil were individually processed through a 32×32 high-pass filter once to remove contributions from field in homogeneity with low spatial frequencies, while high frequency information, mostly produced by local changes, were kept for the resource of additional contrast; 2) processed k-space complex data from each channel were Fourier transformed and a modulus weighted method was employed to combine images from 12 channels to generate a final complex image (after Fourier transformation, complex data from each channel was multiplied by its modulus, summed over all 12 channels, and then divided by the sum of 12 modulus); 3) from the final complex image obtained from step 2, a pair of magnitude and phase images were created, the phase images were also called high pass filter corrected phase images because the high pass filter was used; 4) a normalized phase mask was calculated from each corrected phase image and multiplied with the magnitude image four times to produce the final SWI image [12].

### Imaging analysis

Low signal intensity of microvenous structures and hemorrhage within tumors on SWI were defined as intratumoral susceptibility signal intensity (ITSS) [11]. Two genitourinary radiologists with more than 5 years of experience, who were blinded to histopathology diagnosis, independently evaluated the characteristics of ITSS on a commercial workstation (Syngo, Siemens Healthcare, Erlangen, Germany). In ambiguous cases, both reviewers reached a consensus in their decision. Intratumoral calcifications were excluded if the lesions showed hyperintensity in phase imaging [15].

The presence or absence of ITSSs in each tumor was evaluated. A 4-point confidence level scale was used to score the likelihood of presence of ITSS on SWI (0 =  no ITSS, 1 =  a focus of ITSS less than 0.5 cm in the largest dimension in axial planes, 2 =  a focus of ITSS 0.5–1.0 cm in the largest dimension, 3 =  a focus of ITSS greater than 1.0 cm in the largest dimension).

The morphology of the ITSS was classified into two categories: hemorrhage and mircovessels. Hemorrhage was defined as dots with or without conglomeration that were larger than 0.5 cm in diameter [16]. Mircovessels were defined as fine linear structures with or without conglomeration in continuous slices within a tumor. The dominant structure of ITSS was judged with a 4-point confidence level scale (0 = no ITSS in the tumor on SWI; 1 = prominently hemorrhage in the tumor on SWI; 2 = hemorrhage and microvessels approximately equally present in the tumor on SWI; 3 = prominently microvascular structure).

The ratio of ITSS area to tumor area was scored by a 3-point grading system: 0 =  no ITSS, 1 =  the ratio is less than half of the tumor on any slice, 2 = the ratio is greater than half of the tumor in at least one slice.

### Histological analysis

Histopathology was obtained from all masses by radical nephrectomy. All tissue specimens were evaluated retrospectively by a pathologist with 10 years of clinical experience. According to the Fuhrman criteria [17], all cases were categorized into four grades (Grade I–IV). On the basis of the Fuhrman nuclear grade, all cases were merged into a low- (Grade I+II) or high-grade group (Grade III+IV) [18].

### Statistical analyses

Statistical analysis was performed using SPSS (version 17.0; SPSS, Chicago, IL, USA). Results were presented as mean±SD. Nonparametric Mann-Whitney test was used to compare the differences in the likelihood of the presence of ITSS, the dominant structure of ITSSs and the ratio of ITSS area on SWI between low- and high-grade tumors. A difference of *P*<0.05 was considered significant.

## Results

A total of 37 lesions in 37 patients were included in the statistical analysis. All patients were classified into Grade I (12/37), II (14/37), III (8/37) and IV (3/37). There were 26 cases in the low-grade group and 11 cases in the high-grade group.

ITSSs were seen in 33 of 37 patients. No ITSSs were seen in 4 of 37 patients with low-grade tumors. There was no significant difference in the likelihood of the presence of ITSS on SWI between the low and high group (*P*>0.05). Mean scores of the likelihood of the presence of ITSS were 1.77±1.07 for low-grade CRCCs and 2.64±0.67 for high-grade CRCCs, respectively.

**Figure 1:**
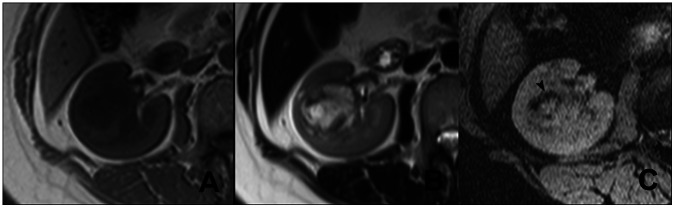
ITSS in a case of CRCCs (Grade II). In the right kidney, a mass appears heterogeneous signal intense on T1WI (A) and T2WI (B). No significant hemorrhage is seen in the tumors. On SWI (C), multiple fine linear structures with partial conglomeration are seen (arrow head), which represent intratumoral microvessels.

**Figure 2:**
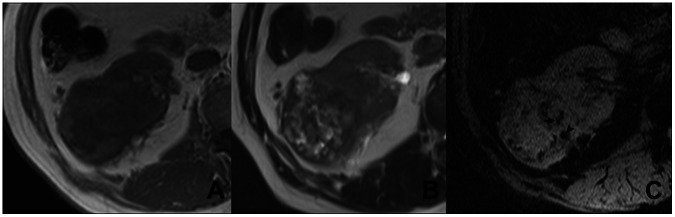
ITSS in a case of CRCCs (Grade III). In the right kidney, a round mass with heterogeneous signal intense is seen on T1WI (A) and T2WI (B). Some necrotic areas are seen. On SWI (C), dot-like (arrow head) and linear (arrow) ITSSs are simultaneously present, which respectively represent intratumoral hemorrhage and micro vessels.

The dominant structure of the ITSSs on SWI is listed in Table 1. Mean scores of dominant structures of ITSSs and the ratio of ITSS area to tumor area on SWI are listed in Table 2. Mean scores of dominant structures of ITSSs on SWI were significantly higher for low-grade CRCCs (Fig. 1) than that for the high-grade CRCCs (Fig. 2) (*P*<0.005). The ratio of ITSS area to tumor area on SWI was significantly higher for the high-grade group than that for the low-grade group (*P*<0.005).

**Table 1:** The dominant morphology of ITSSs in CRCCs.

	Grade I (n = 12)	Grade II (n = 14)	Grade III (n = 8)	Grade IV (n = 3)
No ITSSs	3	1	0	0
Hemorrhage (Dots with or without conglomeration)	0	0	5	3
Microvessels (Fine linear structures with or without conglomerated)	4	7	0	0
Hemorrhage and microvessels almost equally presented in the tumor	5	6	3	0

**Table 2:** Difference in mean scores of ITSSs between low- and high-grade CRCCs.

	Low-grade	High-grade	*P* value
Dominant structures of ITSSs in CRCCs	2.15±1.05	1.27±0.47	<0.005
The ratio of ITSS area to tumor area	0.88±0.43	1.55±0.52	<0.005

## Discussion

The importance of evaluating CRCC grade before treatment is well recognized. Radiofrequency ablation (RA) and active surveillance are nowadays accepted as optional treatment approaches for RCC [19], [20]. Sung et al. [21] reported that there was no significant difference in estimated glomerular filtration rates and three-year recurrence-free survival rates between patients who underwent RA and those who had open partial nephrectomy. The histopathological feature of CRCC is a crucial factor in determining whether RA or active surveillance is optional during the treatment process [22]. Fuhrman grade classification can help determine histopathological features of CRCCs and predict the prognosis of patients based on the microscopic morphology of a neoplasm.

In this study, we demonstrated the potential usefulness of ITSS in grading CRCCs using SWI and revealed that there were significant differences in dominant structure of ITSS and ratio of ITSS area to tumor area between low- and high-grade CRCCs. Mean scores of dominant structures of ITSSs on SWI were significantly lower for high-grade CRCCs (1.27±0.47) than for the lower-grade CRCCs (2.15±1.05). The ratio of ITSS area to tumor area on SWI was significantly higher for the high-grade group (1.55±0.52) than that for low-grade group (0.88±0.43). Our results showed that SWI could be a noninvasive tool for grading CRCCs.

Our results were consistent with previous studies in the brain. It has reported that intratumoral hemorrhage and microvascularity were correlated with pathological grades of gliomas [23], [24]. Park et al. first defined ITSS in their study where they graded gliomas depending on ITSS characters [10]. In their study, ITSSs were seen only in high-grade gliomas. Conglomerated mixed fine linear and dot-like ITSSs were most frequent in gliomas with grade IV, and the fine linear ITSSs were seen in gliomas with grade III. In our study, ITSSs were seen in all patients except 4 patients with low-grade CRCCs. This detail difference between our results and previous studies on grading gliomas may be caused by differences in biological and biochemical behavior between the two types of tumors.

In this study, ITSSs were evaluated and categorized into two different categories: hemorrhage and microvessels. The conventional MRI appearances of hemorrhage relate with the onset time of hemorrhage. SWI is exquisitely sensitive to blood products and can detect more hemorrhagic lesions at different stages than T1WI and T2WI [25]. In a previous study, Xing et al. [14] also demonstrated that SWI can detect intratumoral hemorrhage more accurately and sensitively than non-contrast enhanced MRI. Our results demonstrated that more intratumoural vascular structures and less hemorrhage were present in low-grade tumors than those in high-grade CRCCs. Some previous studies using immunohistochemical methods have also reported that the number of microvessels in high-grade CRCCs was significantly lower than in low-grade CRCCs and correlated with CRCC grades [26], [27]. Our key conclusion of this study, i.e. the dominant structure of ITSSs on SWI was helpful in grading CRCCs, is consistent with the immunohistochemical results.

There were several limitations in our study. First, all selected patients underwent radical nephrectomy and this was a retrospective study in nature. Second, there were only a few patients with high-grade CRCCs. Third, we only evaluated the value of SWI on grading CRCCs in this study while other subtypes of RCC was not evaluated.

In conclusion, our study suggests that SWI is a useful technique to analyze the structural characteristics of CRCCs and grade CRCCs by dominant structures of ITSS and the ratio of ITSS area to tumor area.
